# Trichostatin A Modulates Thiazolidinedione-Mediated Suppression of Tumor Necrosis Factor α-Induced Lipolysis in 3T3-L1 Adipocytes

**DOI:** 10.1371/journal.pone.0071517

**Published:** 2013-08-09

**Authors:** Juu-Chin Lu, Yu-Tzu Chang, Chih-Tien Wang, Yu-Chun Lin, Chun-Ken Lin, Zhong-Sheng Wu

**Affiliations:** 1 Department of Physiology and Pharmacology, College of Medicine, Chang Gung University, Taoyuan, Taiwan; 2 Graduate Institute of Biomedical Sciences, College of Medicine, Chang Gung University, Taoyuan, Taiwan; 3 Department of Biomedical Sciences, College of Medicine, Chang Gung University, Taoyuan, Taiwan; 4 Institute of Molecular and Cellular Biology, National Taiwan University, Taipei, Taiwan; The University of New South Wales, Australia

## Abstract

In obesity, high levels of tumor necrosis factor α (TNFα) stimulate lipolysis in adipocytes, leading to hyperlipidemia and insulin resistance. Thiazolidinediones (TZDs), the insulin-sensitizing drugs, antagonize TNFα-induced lipolysis in adipocytes, thereby increasing insulin sensitivity in diabetes patients. The cellular target of TZDs is peroxisome proliferator-activated receptor γ (PPARγ), a nuclear receptor that controls many adipocyte functions. As a transcription factor, PPARγ is closely modulated by coregulators, which include coactivators and corepressors. Previous studies have revealed that in macrophages, the insulin-sensitizing effect of PPARγ may involve suppression of proinflammatory gene expression by recruiting the corepressor complex that contains corepressors and histone deacetylases (HDACs). Therefore, we investigated whether the corepressor complex is involved in TZD-mediated suppression of TNFα-induced lipolysis in 3T3-L1 adipocytes. Trichostatin A (TSA), a pan HDAC inhibitor (HDACI) that inhibits class I and II HDACs, was used to examine the involvement of HDACs in the actions of TZDs. TSA alone increased basal lipolysis and attenuated TZD-mediated suppression of TNFα-induced lipolysis. Increased basal lipolysis may in part result from class I HDAC inhibition because selective class I HDACI treatment had similar results. However, attenuation of TZD-mediated TNFα antagonism may be specific to TSA and related hydroxamate-based HDACI rather than to HDAC inhibition. Consistently, corepressor depletion did not affect TZD-mediated suppression. Interestingly, TSA treatment greatly reduced PPARγ levels in differentiated adipocytes. Finally, extracellular signal-related kinase 1/2 (ERK1/2) mediated TNFα-induced lipolysis, and TZDs suppressed TNFα-induced ERK phosphorylation. We determined that TSA increased basal ERK phosphorylation, and attenuated TZD-mediated suppression of TNFα-induced ERK phosphorylation, consistent with TSA’s effects on lipolysis. These studies suggest that TSA, through down-regulating PPARγ, attenuates TZD-mediated suppression of TNFα-induced ERK phosphorylation and lipolysis in adipocytes.

## Introduction

Obesity is characterized by increased proinflammatory cytokine secretion from hypertrophied adipocytes and infiltrated macrophages as well as elevated levels of circulating free fatty acids (FFAs), primarily resulting from lipolysis of triglycerides (TG) stored in adipocytes. Elevated proinflammatory cytokine and FFA levels mediate obesity-associated diseases, such as insulin resistance, type 2 diabetes, and cardiovascular diseases [1], [2]. Tumor necrosis factor α (TNFα) is one of the elevated inflammatory factors in obesity that is elevated and plays an important role in obesity-associated diseases [3], [4]. In addition to its role in inflammation, TNFα also increases lipolysis in adipocytes, which may contribute to elevated FFA circulation [3], [5], [6], [7].

The mechanism by which TNFα stimulates lipolysis is not completely understood. Unlike the acute lipolysis that is stimulated by catecholamines during fasting (within minutes), TNFα requires a longer duration (6-16 hours) to induce measurable lipolysis [8], [9], suggesting that transcriptional regulation is involved [10]. The early signaling pathways that is involved in TNFα-induced lipolysis have been studied in both human and rodent adipocytes. In human adipocytes, p44/42 extracellular signal-related kinase 1/2 (ERK1/2) and c-Jun N-terminal kinase (JNK), but not p38 mitogen-activated protein kinase (MAPK), mediate TNFα-induced lipolysis [10], [11]. By contrast, ERK but not JNK mediates TNFα-induced lipolysis in 3T3-L1 adipocytes [12]. Moreover, elevated cyclic AMP (cAMP) levels and protein kinase A (PKA) activation mediate in TNFα-induced lipolysis in human adipocytes, [7], [13], whereas the involvement of cAMP and PKA in TNFα-induced lipolysis is controversial in mouse adipocytes [12], [14]. Finally, TNFα-induced down-regulation of perilipin, which is a surface protein that protects stored TG in adipocyte lipid droplets from hydrolytic lipase activity, has been observed in both human and murine adipocytes [11], [12].

The insulin-sensitizing drug thiazolidinediones (TZDs), which include rosiglitazone (Rosi) and pioglitazone, have been shown to block TNFα-stimulated lipolysis [8], [12]. TZDs suppress TNFα-induced ERK phosphorylation [12], and reverse TNFα-induced down-regulation of perilipin [8], [12], [15]. However, the detailed mechanism remains incompletely understood. The cellular target of TZDs is peroxisome proliferator-activated receptor γ (PPARγ), which is a nuclear receptor that is modulated by transcriptional coregulators including coactivators and corepressors. The corepressor complex, which includes corepressors and histone deacetylases (HDACs), mediates the PPARγ antagonism against inflammatory gene expression in macrophages [16]. However, the role of corepressors and HDACs in adipocytes remains largely unknown. In particular, whether the corepressors and HDACs are involved in TZD-mediated suppression of TNFα actions, such as lipolysis, remains to be determined.

HDACs can be divided into groups based on homology to yeast HDACs [17]. Classical HDACs are zinc-dependent enzymes which include class I (HDAC1, -2, -3, and -8) and class II HDACs (HDAC4, -5, -6, -7, -9, -10). Class I HDACs generally localize to the nucleus, whereas class II HDACs can shuttle between the nucleus and the cytoplasm. HDACs have been recognized to play an important role in regulating proliferation, differentiation, and development [18]. HDAC inhibitors (HDACIs) have been developed as therapeutic drugs for treating cancer and certain neurodegenerative disorders [17]. The hydroxamate-based HDACI vorinostat (also known as suberoylanilide hydroxamic acid, SAHA) is currently approved for cutaneous T-cell lymphoma treatment [19], whereas the aliphatic acid-based HDACI valproic acid (VPA) has long been used for epilepsy or bipolar disorder treatment [20]. While HDACIs demonstrate great promise for disease treatment, the mechanisms by which these effects are mediated remain elusive, and side effects of HDACI treatment have been reported including metabolic effects [21], [22]. For example, VPA treatment has been associated weight gain and insulin resistance in patients [21], [23]. The pan HDACIs such as SAHA and its related compound trichostatin A (TSA) inhibit both class I and class II HDACs, whereas VPA has high potency for class I HDACs. Moreover, selective HDACIs, such as MS275 (class I) and MC1568 (class II), have also been developed. In preadipocytes, HDACI treatment either suppresses [24], [25], [26] or promotes [27], [28] adipocyte differentiation. However, the effects of HDACIs on differentiated adipocyte physiology such as lipolysis have not been examined.

In the current study, we used TSA, a natural hydroxamic acid and a pan HDACI, to assess the involvement of the HDACs in TZD-mediated suppression of TNFα-induced lipolysis in 3T3-L1 adipocytes. We observed that TSA treatment not only increased basal lipolysis, but also attenuated TZD-mediated suppression of TNFα-induced lipolysis in adipocytes. Increased basal lipolysis by TSA may be in part due to inhibition of class I HDACs, whereas attenuation of TZD-mediated suppression of TNFα action may be specific to TSA and its related hydroxamate-based HDACI SAHA. Moreover, TSA treatment greatly down-regulated PPARγ isoforms in differentiated adipocytes, thereby affecting TZD-mediated suppression of TNFα-induced ERK phosphorylation and lipolysis. Given that SAHA is currently approved for clinical use, the detailed mechanisms underlying HDACI-mediated cellular modulation merit further investigation.

## Materials and Methods

### Chemicals and Reagents

TSA (T8852), SAHA (SML0061), MC1568 (M1824), and U0126 (U120) were purchased from Sigma Chemical (St. Louis, MO). MS275 (E-3866) was purchased from LC Laboratories (Woburn, MA). Recombinant murine TNFα (No. 410-MT) was from R & D Systems (Minneapolis, MN). Rosiglitazone (No.71740) was from Cayman Chemical (Ann Arbor, MI). Polyclonal antibodies against phospho-ERK1/2 (Thr202/Tyr204, #4377), total ERK1/2 (#9102), and rabbit monoclonal antibodies against PPARγ (clone 81B8, #2443) were from Cell Signaling Technology (Beverly, MA). Anti-acetyl-Histone H3 (#06-599) and anti-NCoR (#ABE251) antibodies were from Millipore Corporation (Temecula, CA). Anti-SMRT antibodies (#PA1-842) were from Thermo Scientific (Rockford, IL). Anti-α-tubulin (#T5168) and anti-acetylated tubulin (#T6793) antibodies were from Sigma Chemical (St Louis, MO).

### Cell Culture and Differentiation

3T3-L1 fibroblasts (CL-173) were obtained from American Type Culture Collection (Manassas, VA), and were cultured and differentiated as described previously [29]. In brief, cells were cultured in growth media (Dulbecco’s modified Eagle medium with 4.5 g/L glucose, 10% fetal bovine serum, 1% glutamine, and 0.5% penicillin/streptomycin). Differentiation was induced in post-confluent cells with growth media containing 500 µM isobutylmethylxanthine, 0.2 µM dexamethasone, and 2.5 µg/ml insulin for 3-4 days, and cells were replenished with growth media every 3-4 days. Experiments were performed in adipocytes 12-16 days post differentiation.

### Electroporation and siRNA

Differentiated 3T3-L1 adipocytes were electroporated at 200 V and 950 µF with 2 nmole siRNA using a Gene Pulser Xcell electroporator (Bio-Rad, Hercules, CA) and were plated onto appropriate plates or dishes for experiments. Experiments were performed 48 h after electroporation. siRNA duplexes were designed using either the published sequences or a commercial design program (Thermo Scientific). The siRNA sequences were as follows: PPARγ, 5′-CAA CAG GCC TCA TGA AGA A-3′ [30], 5′-ATT AAG GAA TTC ATG TCG TAG-3′ [31]; NCoR, 5′-GCT GCA TCC AAG GGC CAT G-3′ [32], 5′-GGG CAA AGC TAT TTA GGA A-3′; SMRT, 5′-AAG CTG AAG AAG AAG CAG CAA-3′ [33], 5′-AGA CCA TCA TCA ATG ACT A-3′. Luciferase siRNA duplex 5′-TCG AAG TAT TCC GCG TAC G-3′ were used as a control. The absence of homology to any other gene was confirmed using a BLAST search (National Center for Biotechnology Information, National Institutes of Health).

### RNA Analysis

Total cellular RNA was isolated and purified using TRIzol reagent (Ambion, Austin, TX) according to the manufacturer’s instructions. First strand cDNA was synthesized from 1 µg total RNA using high-capacity cDNA RT kits (Applied Biosystems). SYBR green PCR was performed using the MiniOpticon real-time PCR detection system (Bio-Rad, Hercules, CA). The following primers were used for PCR: PPARγ forward 5′-GCC CTT TGG TGA CTT TAT GG-3′, reverse 5′-CAG CAG GTT GTC TTG GAT GT-3′; SMRT forward 5′-GGG AGT GAA CGG TCT CAG GAG C-3′, reverse 5′-GTA GTA GCT CCA GGC GGG GG-3′; NCoR forward 5′-TTA CCA CAG GCA GAC ACC AG-3′, reverse 5′-CCG TAT GGT CAG AGG GTT GT-3′. 36B4 forward 5′-GCG ACC TGG AAG TCC AAC TAC-3′, reverse 5′-ATC TGC TGC ATC TGC TTG G-3′. Gene expression levels were calculated after normalization to the housekeeping gene 36B4 using the ΔΔCT method as described by the manufacturer and expressed as relative mRNA levels compared with the control.

### Measurement of Lipolysis

Lipolysis was measured using free glycerol reagent (Sigma, St Louis, MO) according to manufacturer’s specifications. In brief, 3T3-L1 adipocytes were washed twice with PBS and were then incubated in 0.5 ml phenol red-free DMEM containing 3% BSA and the desired treatments for 24 h. Cell media were collected and centrifuged at full speed for 1 min to remove cell debris, and the supernatants were divided into aliquots for the assays. Ten microliters of sample was incubated with 180 µl free glycerol assay reagent for 15 min at room temperature, and the absorbance was read at 540 nm. A standard curve constructed from the glycerol standards was used to calculate glycerol concentration in the culture supernatants. The cells remaining on the plate were washed and lysed in 1 N NaOH, and protein concentrations were measured and used to normalize glycerol release.

### Western Blot Analysis

Western blot analysis was performed as described previously [34]. In brief, cells were extracted with RIPA lysis buffer (50 mM HEPES, pH 7.4, 1% NP-40, 150 mM NaCl, 1 mM EDTA, 1 mM phenylmethylsulfonyl fluoride, 1 µg/ml leupeptin, 1 mM sodium orthovanadate, 1 mM sodium fluoride). Twenty microliters of cellular protein lysate was electrophoresed through standard Laemmli SDS polyacrylamide gels (7-12% gels), transferred to polyvinylidene fluoride membranes, and then probed with appropriate antibodies. Membranes were blocked for 1 h in 5% BSA in TBST (100 mM Tris-HCl, pH 7.5, 150 mM NaCl, and 0.1% Tween 20) and then incubated in primary antibodies at 4°C overnight. Membranes were washed three times with TBST and then incubated with secondary antibodies in 5% milk in TBST at room temperature for 1 h. Membranes were washed three times with TBST, and then signals were visualized by enhanced chemiluminescence followed by autoradiography.

### Statistics

All of the data were presented as the mean ± S.E. Differences between the means of two groups were evaluated for statistical significance with paired or unpaired Student’s two-tailed *t*-tests. A *p* value cut-off of 0.05 was considered statistically significant (InStat 3, GraphPad).

## Results

### TSA Treatment Reduces Rosi-mediated Suppression of TNFα-induced Lipolysis in 3T3-L1 Adipocytes

**Figure 1:**
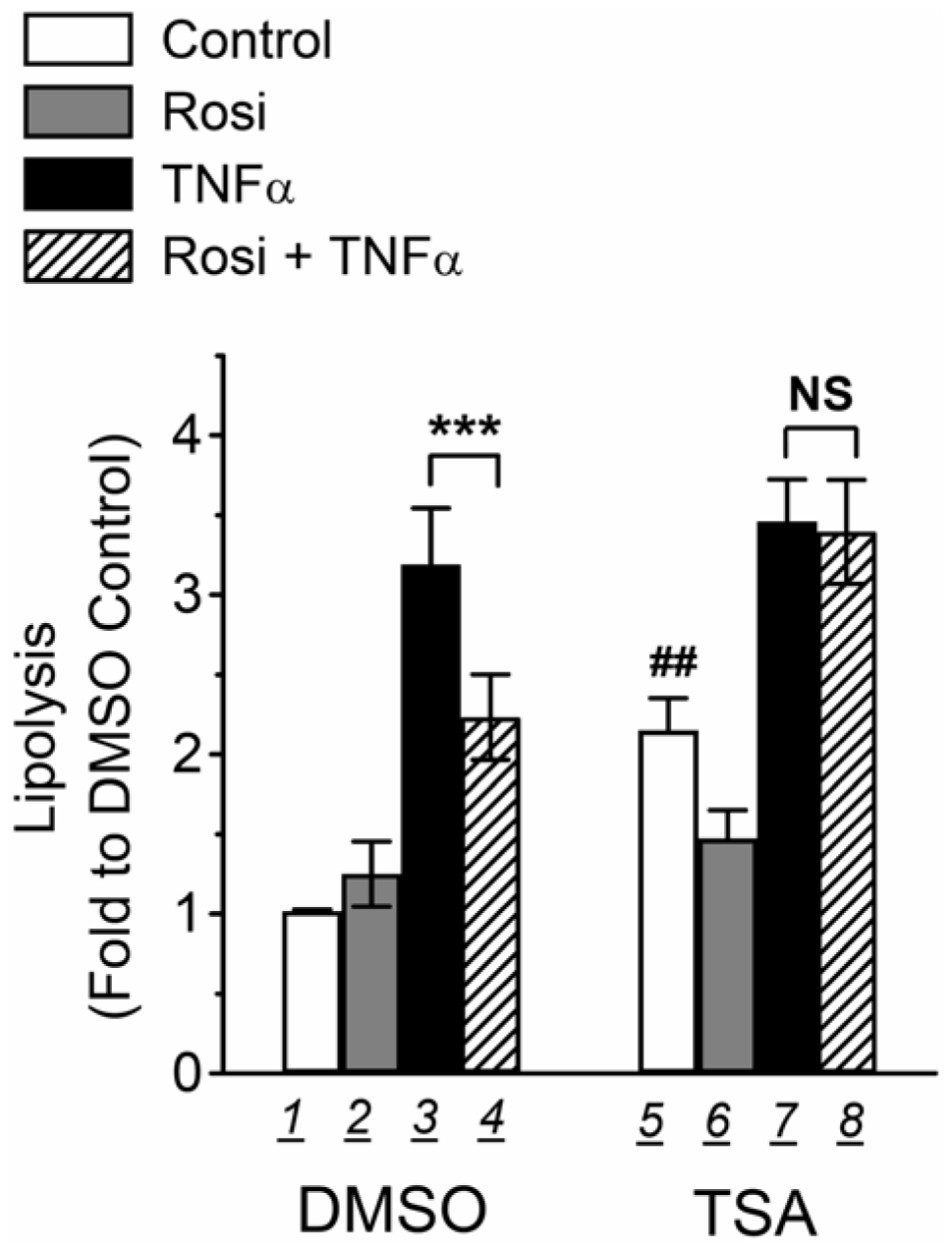
TSA treatment attenuates Rosi suppression of TNFα-induced lipolysis in 3T3-L1 adipocytes. 3T3-L1 adipocytes were treated with vehicle (Control), 1 µM Rosi (Rosi), 10 ng/ml TNFα (TNFα), or both (Rosi+TNFα), together with vehicle (DMSO, bars 1-4) or 660 nM TSA (TSA, bars 5-8) for 24 h. Glycerol released into the media and protein concentrations of cell lysate were determined as described in *Materials and Methods*. Each point represents the mean ± S.E. of seven independent experiments. Asterisks denote significant differences (***p<0.001). NS, not significant. ^##^p<0.01 bar 5 vs 1.

Chronic TNFα treatment increases lipolysis in 3T3-L1 adipocytes, whereas Rosi suppresses the TNFα-induced lipolysis [8], [12]. In macrophages, a complex of transcription corepressor and HDACs has been shown to mediate Rosi suppression of inflammatory gene expression [35], [36]. Since TNFα is a proinflammatory cytokine which induces many proinflammatory actions such as inflammatory gene expression and proinflammatory signaling pathways, we tested if anti-inflammatory mechanism may be involved in Rosi-mediated suppression of TNFα-induced lipolysis in adipocytes. To determine whether HDACs may be involved in Rosi-mediated suppression of TNFα-induced lipolysis, differentiated 3T3-L1 adipocytes were treated with or without 200 ng/ml (equal to 660 nM) TSA, a pan HDAC inhibitor, for 24 h, and its effects on Rosi-mediated suppression of TNFα-induced lipolysis were determined by measuring glycerol content in the media after treatment. As shown in Fig. 1, TNFα treatment induced a 3-fold increase of lipolysis in adipocytes (bar 3 vs 1), whereas co-treatment with 1 µM Rosi suppressed TNFα-induced lipolysis (bar 4 vs 3), which is consistent with previous reports [12]. Interestingly, TSA treatment alone elevated basal lipolysis (bar 5 vs 1). Moreover, TSA treatment also attenuated Rosi-mediated suppression of TNFα-induced lipolysis (bar 8 vs 7).

**Figure 2:**
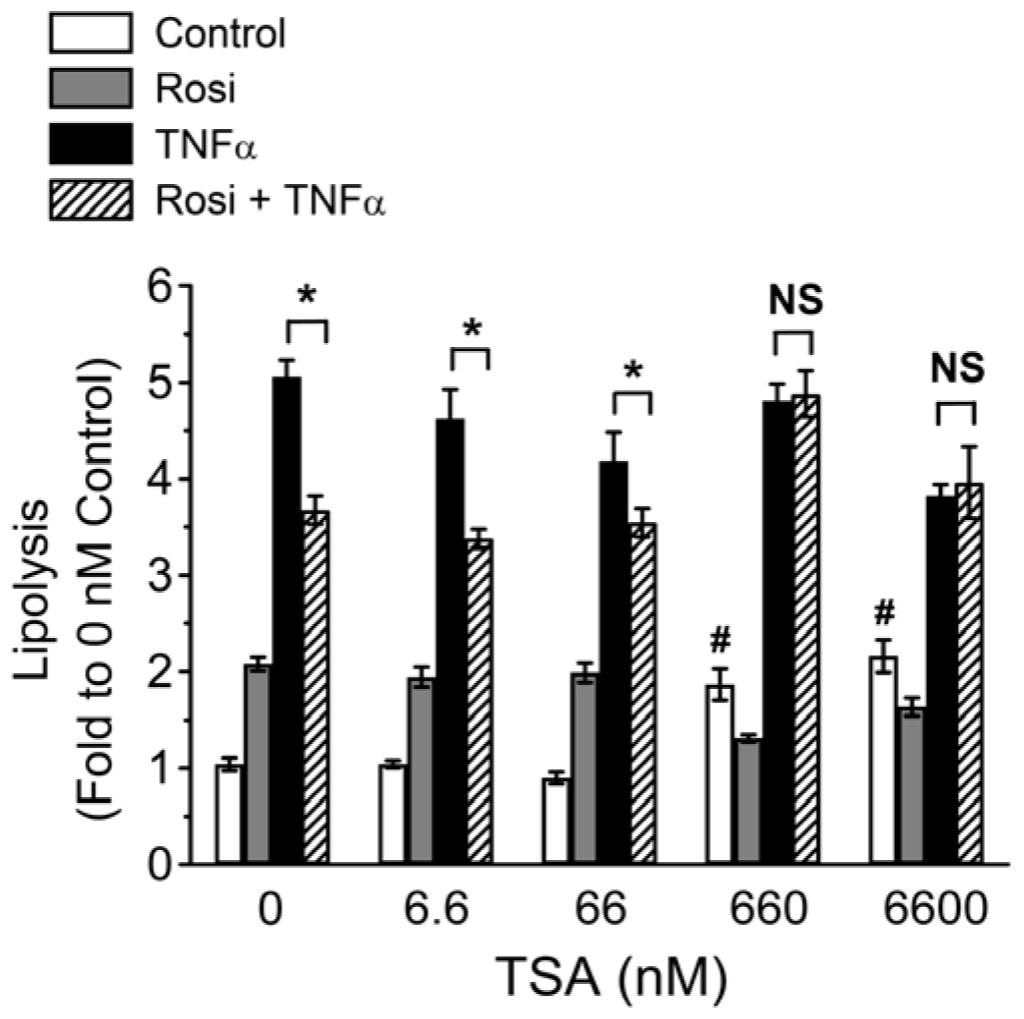
TSA attenuates Rosi-mediated suppression of TNFα-induced lipolysis in a dose-dependent manner. 3T3-L1 adipocytes were treated with vehicle (Control), 1 µM Rosi (Rosi), 10 ng/ml TNFα (TNFα), or both (Rosi+TNFα), together with increasing TSA doses (0, 6.6, 66, 660, 6600 nM) for 24 h. Glycerol released into the media and protein concentrations of cell lysate were determined as described in *Materials and Methods*. Each point represents the mean ± S.E. of three independent experiments. Asterisks denote significant differences (p<0.05). NS, not significant. ^#^p<0.05 vs 0 nM control.

To determine the effective TSA dose that attenuated Rosi-mediated suppression of TNFα-induced lipolysis, a dose response experiment was performed. 3T3-L1 adipocytes were treated with different TSA doses (0, 6.6, 66, 660, 6600 nM), and TNFα-induced lipolysis was measured in the presence or absence of Rosi. As shown in Fig. 2, TSA-mediated effects on basal lipolysis and attenuation of TZD action were dose-dependent: TSA was effective at 660 nM or greater, but the effect was gradually lost when the concentrations dropped to 66 nM or lower.

### PPARγ, but not its Corepressor NCoR or SMRT, is Required for Rosi-mediated Suppression of TNFα-induced Lipolysis

**Figure 3:**
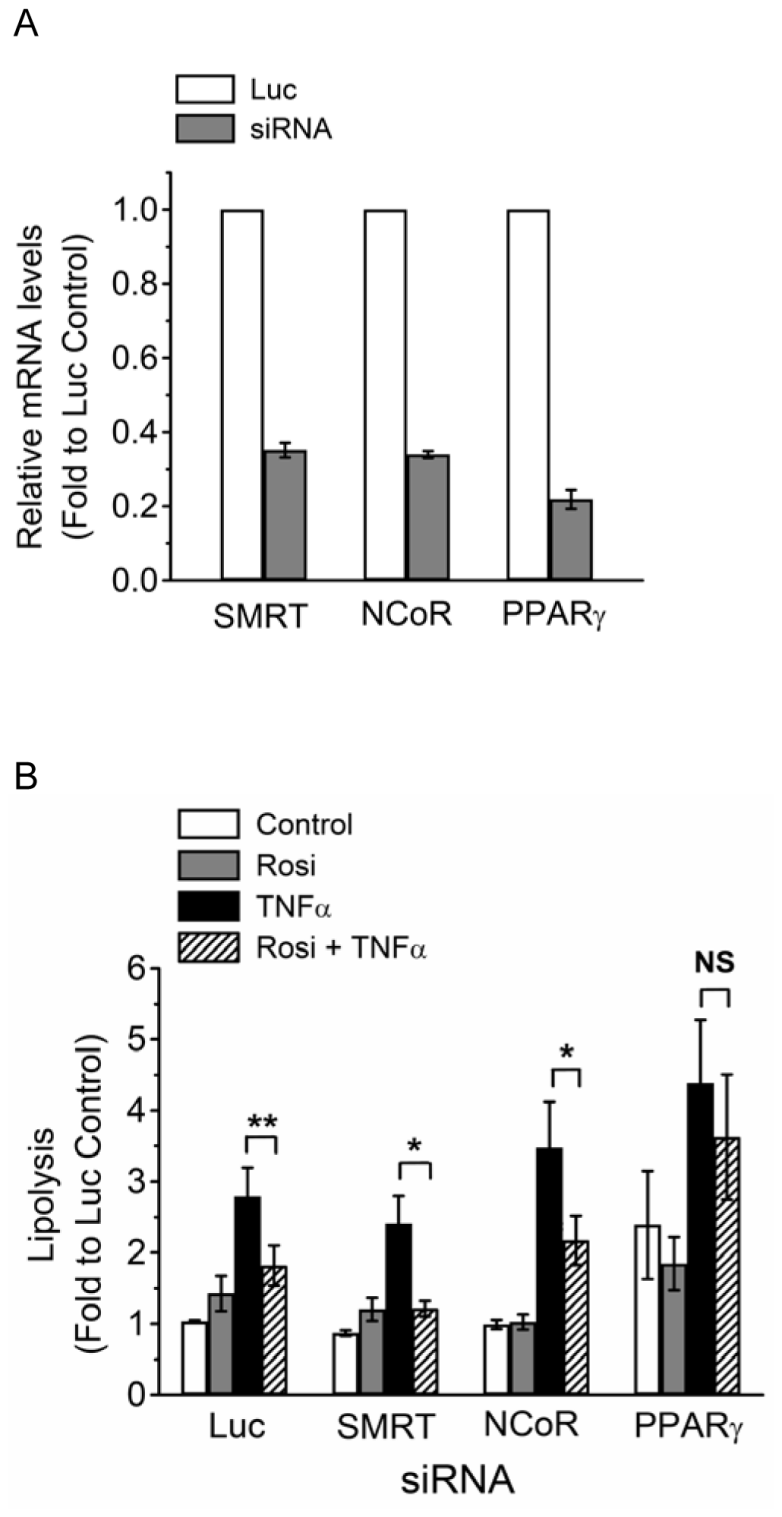
PPARγ, but not corepressor SMRT or NCoR, is required for Rosi-mediated suppression of TNFα-induced lipolysis. (A) 3T3-L1 adipocytes were transfected with non-targeting luciferase siRNA (Luc), or siRNA against SMRT, NCoR, or PPARγ. The levels of mRNA were determined by qPCR. Each point represents the mean ± S.E. of at least three independent experiments. (B) 3T3-L1 adipocytes were transfected with control (Luc), SMRT, NCoR, or PPARγ siRNA. 24 h post transfection, cells were treated with vehicle (Control), 1 µM Rosi (Rosi), 10 ng/ml TNFα (TNFα), or both (Rosi+TNFα) for additional 24 h. Glycerol released into the media and protein concentrations of cell lysate were determined as described in *Materials and Methods*. Each point represents the mean ± S.E. of four independent experiments. Asterisks denote significant differences (*p<0.05; **p<0.01). NS, not significant.

One potential mechanism by which HDACs modulate transcription factor action is through transcriptional corepressors, which recruit HDACs to the gene promoters for transcriptional suppression. To examine if corepressors may be involved in Rosi-mediated suppression of TNFα-induced lipolysis, the expression of PPARγ corepressors such as nuclear receptor corepressor (NCoR) or silencing mediator of retinoic acid and thyroid hormone receptor (SMRT) was depleted in differentiated 3T3-L1 adipocytes by RNAi-mediated gene silencing. The RNAi knockdown efficiency was determined by both real-time qPCR (Fig. 3A) and Western blot analysis (Fig. S1).

The effect of corepressor depletion on Rosi-mediated suppression of TNFα-induced lipolysis was determined. As shown in Fig. 3B, TNFα induced lipolysis in control adipocytes (Luc), and Rosi co-treatment suppressed this induction, a result similar to what were observed in non-transfected cells in Fig. 1. SMRT or NCoR depletion did not affect Rosi-mediated suppression of TNFα-induced lipolysis (hatched vs black bar, Fig. 3B), suggesting that corepressor SMRT or NCoR may not be involved in Rosi-mediated suppression of TNFα-induced lipolysis. By contrast, PPARγ depletion elevated basal lipolysis, although these data were not significantly different compared to Luc control. Moreover, PPARγ depletion attenuated Rosi-mediated suppression of TNFα-induced lipolysis, confirming the requirement of PPARγ for Rosi-mediated suppression.

### Hydroxamic Acid-based HDACI SAHA, but not Other HDACIs, Attenuates Rosi-mediated Suppression of TNFα-induced Lipolysis in 3T3-L1 Adipocytes

**Figure 4:**
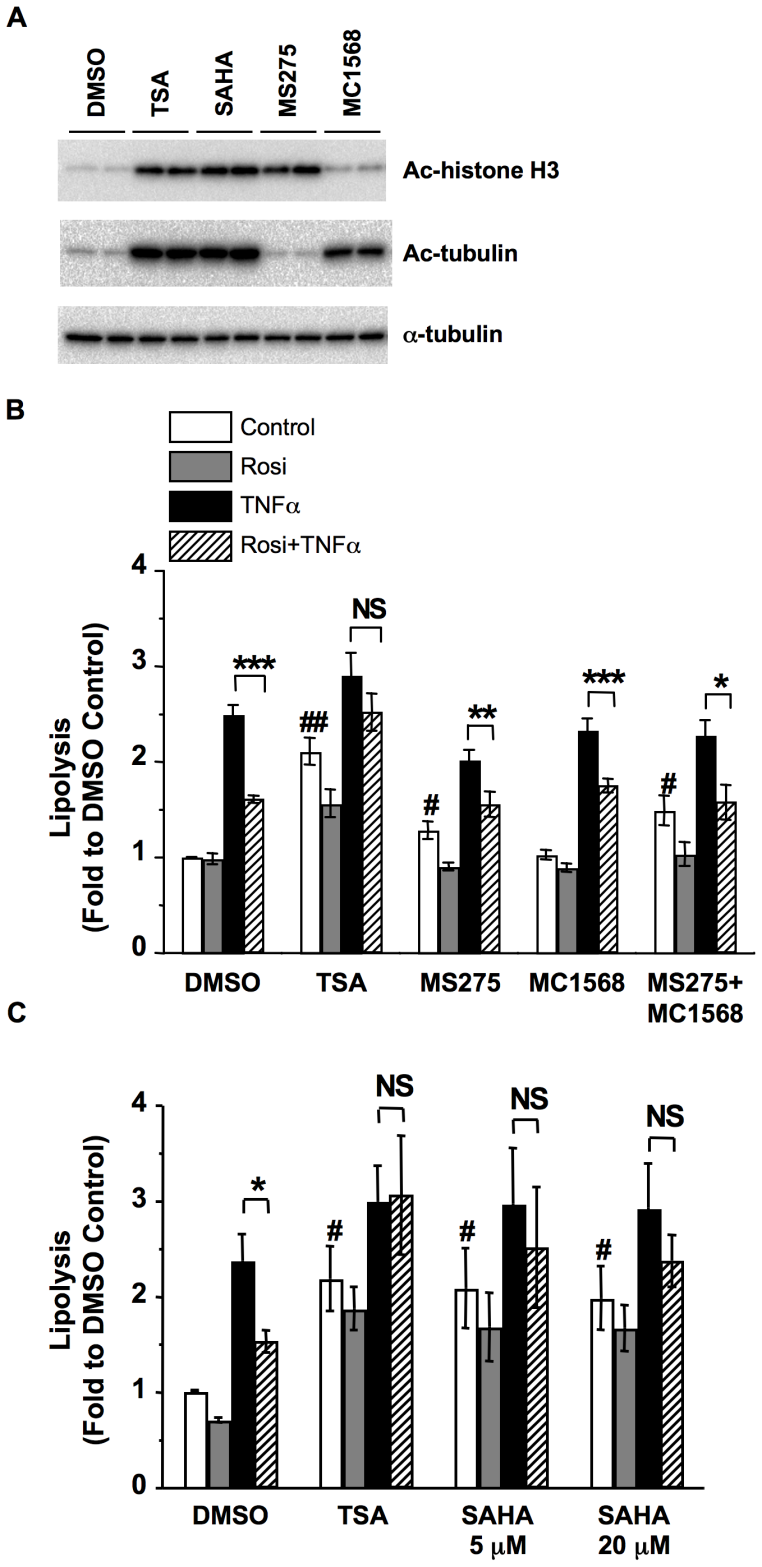
TSA and SAHA, but not other HDACI, attenuates Rosi suppression of TNFα-induced lipolysis. (A) 3T3-L1 adipocytes were treated with DMSO, 660 nM TSA, 20 µM SAHA, 10 µM MS275, or 5 µM MC1568 for 24 h. Cellular proteins were solubilized and subjected to SDS-PAGE and Western blot analysis with the indicated antibodies. Samples were treated in duplicate. Representative immunoblots from three independent experiments are shown. (B) 3T3-L1 adipocytes were treated with vehicle (Control), 1 µM Rosi (Rosi), 10 ng/ml TNFα (TNFα), or both (Rosi+TNFα), together with vehicle (DMSO), 660 nM TSA, 10 µM MS275, 5 µM MC1568, or combination of MS275 and MC1568 (MS275+MC1568) for 24 h. (C) 3T3-L1 adipocytes were treated with vehicle (Control), 1 µM Rosi (Rosi), 10 ng/ml TNFα (TNFα), or both (Rosi+TNFα), together with vehicle (DMSO), 660 nM TSA (TSA), 5 or 20 µM SAHA (SAHA) for 24 h. Glycerol released into the media and protein concentrations of cell lysate were determined as described in *Materials and Methods*. Each point represents the mean ± S.E. of four independent experiments. Asterisks denote significant differences (*p<0.05; **p<0.01; ***p<0.001). NS: not significant. ^#^p<0.05, ^##^p<0.01 vs the corresponding DMSO Control.

TSA is a broad-spectrum HDACI that inhibits class I and II HDAC activities. To determine which class of HDACs may be involved in TSA-mediated attenuation of Rosi-mediated suppression of TNFα-induced lipolysis, we treated the cells with selective HDACIs and examined their effects on Rosi-mediated suppression of TNFα-induced lipolysis. To confirm the selectivity of these HDAC inhibitors, we first examined the hyperacetylation of histone H3 and tubulin, which are substrates of class I and class II HDACs, respectively. As shown in Fig. 4A, the broad-spectrum HDACI TSA and its related hydroxamate-based HDACI SAHA increased acetylation of both histone H3 and tubulin, consistent with their inhibition of class I and class II HDACs. Selective class I HDACI MS275 treatment increased only acetylated histone H3 but not tubulin. By contrast, class II HDACI MC1568 treatment selectively enhanced acetylated tubulin without increasing histone H3 acetylation.

We then examined the effects of selective HDACI on Rosi-mediated suppression of TNFα-induced lipolysis. As shown in Fig. 4B, TSA treatment increased basal lipolysis and attenuated the Rosi-mediated suppression of TNFα-induced lipolysis, similar to the results shown in Figs. 1 and 2. Similar to TSA, treatment with class I HDACI MS275 also increased basal lipolysis (white bar, MS275 vs DMSO). However, MS275 treatment did not affect Rosi-mediated suppression of TNFα-induced lipolysis (hatched vs black bar). By contrast, class II HDACI MC1568 treatment did not affect basal lipolysis or Rosi-mediated suppression of TNFα-induced lipolysis. These results suggested that the effect of TSA to elevate basal lipolysis is in part due to class I HDAC inhibition. However, TSA-mediated attenuation of Rosi-mediated suppression of TNFα-induced lipolysis may not result from HDAC inhibition. Simultaneously treatment with both MC1568 and MS275 did not affect the Rosi-mediated suppression of TNFα-induced lipolysis (Fig. 4B), ruling out the need to inhibit both class I and class II HDACs for TSA’s modulation on Rosi-mediated suppression.

TSA belongs to the hydroxamate-based HDACIs which also include SAHA, an HDACI currently approved by the FDA for the treatment of cutaneous T-cell lymphoma [19]. To access whether the TSA effects could be reproduced by another hydroxamate-based HDACI, 3T3-L1 adipocytes were treated with vehicle (DMSO), TSA, or SAHA, and TNFα-induced lipolysis was determined in the presence or absence of Rosi. As shown in Fig. 4C, treatment with 5 or 20 µM SAHA also elevated basal lipolysis and attenuated Rosi-mediated suppression of TNFα-induced lipolysis. Together, these results suggest that the effects of TSA treatment on Rosi-mediated suppression of TNFα-induced lipolysis may be specific to hydroxamate-based HDACIs rather than general HDAC inhibition.

### TSA Down-regulates PPARγ Levels in 3T3-L1 Adipocytes

**Figure 5:**
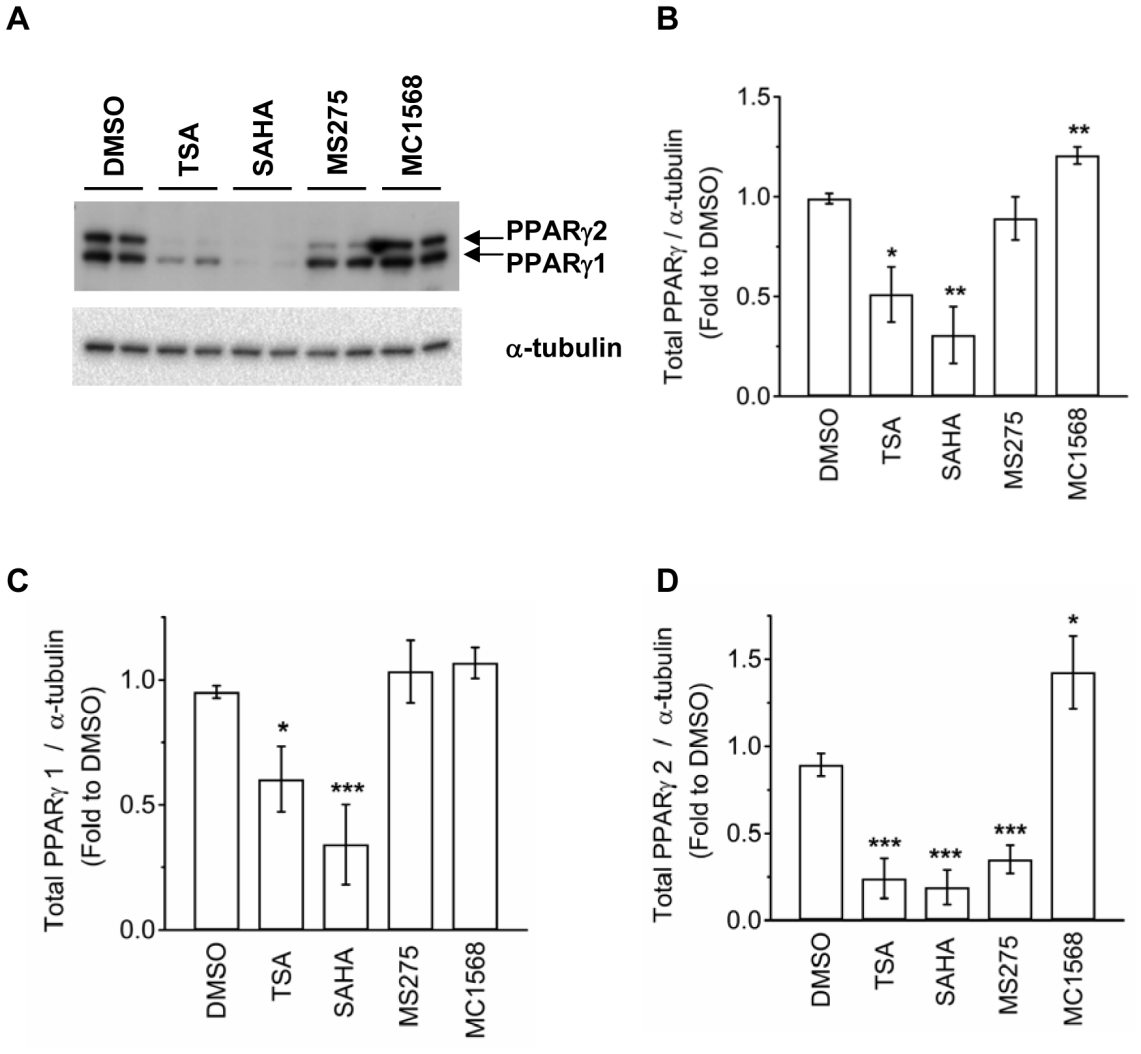
Effects of HDACI treatment on the levels of PPARγ expression. (A) 3T3-L1 adipocytes were treated with DMSO, 660 nM TSA, 20 µM SAHA, 10 µM MS275, or 5 µM MC1568 for 24 h. Cellular proteins were solubilized and subjected to SDS-PAGE and Western blot analysis with the indicated antibodies. Samples were treated in duplicate. Representative immunoblots from three independent experiments were shown in 5A. (B-D) Quantification data for total PPARγ (B), PPARγ1(C), and PPARγ2 (D) are shown. Asterisks denote significant differences compared with DMSO control (*p<0.05; **p<0.01; ***p<0.001).

The absence of effects on Rosi-mediated suppression of TNFα-induced lipolysis by corepressor depletion (Fig. 3) and class-specific HDACI treatment (Fig. 4) suggested that other mechanisms may account for TSA attenuation of the Rosi-mediated suppression of TNFα-induced lipolysis. Previous studies have reported that daily HDACI treatment during preadipocyte adipogenesis reduced PPARγ levels, thereby inhibiting adipocyte differentiation [24]. By contrast, in differentiated adipocytes, treatment with 1 mM VPA did not affect PPARγ expression [37], [38]. However, the effects of other HDACIs on PPARγ in differentiated adipocytes have not been examined. Therefore, differentiated 3T3-L1 adipocytes were treated with vehicle (DMSO), TSA, SAHA, MS275, or MC1568, and the expression of PPARγ1 and γ2 was determined by Western blot analysis. As shown in Fig. 5, PPARγ1 and PPARγ2 levels were greatly reduced in adipocytes that had been treated with broad-spectrum HDACIs TSA or SAHA. Class I HDACI MS275 also reduced the levels of PPARγ2 but not PPARγ1. By contrast, class II HDACI MC1568 treatment slightly increased PPARγ2 or total PPARγ levels without affecting PPARγ1 levels. These results suggested that through inhibition of class I HDACs, TSA treatment greatly reduced PPARγ2 expression in differentiated adipocytes. However, TSA and SAHA, but not class I HDACI MS275 treatment, also reduced PPARγ1 levels. Because TSA and SAHA, but not MS275, attenuated the Rosi-mediated suppression of TNFα-induced lipolysis (Fig. 4B,C), these results suggested that a mechanism by which TSA and SAHA attenuated Rosi-mediated suppression of TNFα-induced lipolysis may result from down-regulation of both PPARγ1 and 2 in differentiated adipocytes.

### Down-regulation of PPARγ by TSA Treatment is Dose- and time- Dependent, and may Involve Proteasomal Degradation

**Figure 6:**
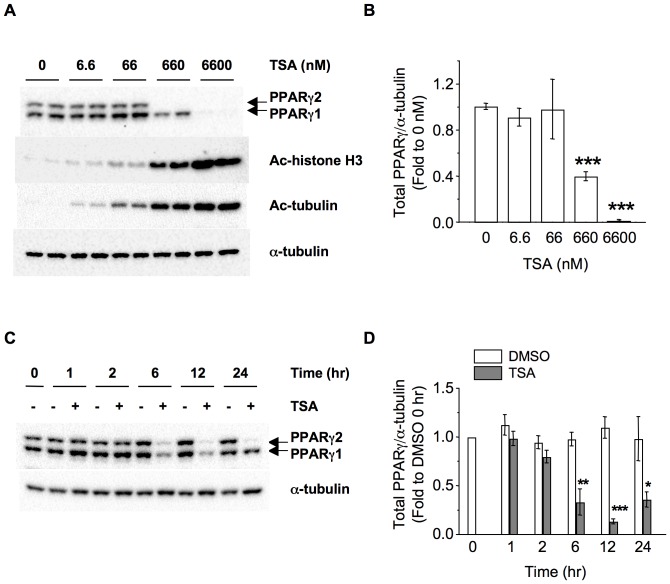
Down-regulation of PPARγ by TSA treatment is dose- and time- dependent. (A, B) 3T3-L1 adipocytes were treated in duplicate with increasing TSA doses (0, 6.6, 66, 660, 6600 nM) for 24 h. Cellular proteins were solubilized and subjected to SDS-PAGE and Western blot analysis with the indicated antibodies. Representative immunoblots from three independent experiments were shown in 6A. Quantification data for total PPARγ are shown in 6B. Asterisks denote significant differences compared with 0 nM control (***p<0.001). (C, D) 3T3-L1 adipocytes were treated with vehicle (DMSO) or 660 nM TSA (TSA) for 0, 1, 2, 6, 12, or 24 h. Cellular proteins were solubilized and subjected to SDS-PAGE and Western blot analysis with the indicated antibodies. Representative immunoblots from four independent experiments were shown in 6C. Quantification data for total PPARγ are shown in 6D. Asterisks denote significant difference compared with corresponding DMSO value at the same time point (*p<0.05; **p<0.01; ***p<0.001).

In Fig. 2, the TSA effects on basal lipolysis and attenuation of Rosi action were dose-dependent. We also found that TSA treatment down-regulated PPARγ levels (Fig. 5). To determine if TSA down-regulation of PPARγ is also dose-dependent, we performed a dose response experiment. Differentiated 3T3-L1 adipocytes were treated with different TSA doses (0, 6.6, 66, 660, 6600 nM) and their effects on PPARγ levels were determined. As expected, the HDACI activity of TSA, determined by hyperacetylation of histone H3 and tubulin, was increased in a dose-dependent manner (Fig. 6A). However, the concentration required for TSA to down-regulate PPARγ isoforms was 660 nM or greater (Fig. 6AB and Fig. S2AB), which correlated well with the concentrations that were effective in modulation of basal and Rosi-mediated suppression of TNFα-induced lipolysis (Fig. 2). Thus, the concentrations required for TSA down-regulation of PPARγ in differentiated adipocytes were in contrast with previous reports in preadipocytes, in which 3 nM TSA could down-regulate PPARγ [24], suggesting different sensitivities in preadipocytes and differentiated adipocytes.

The time response experiment of TSA treatment was also performed. As shown in Figures 6CD and S2CD, TSA down-regulated PPARγ isoforms at time points as early as 6 hr. The short incubation time required for TSA down-regulation of PPARγ prompted us to test if proteasomal degradation may be involved. We applied proteasomal inhibitor MG132 in combination with TSA treatment. While TSA treatment down-regulated the levels of PPARγ isoforms, the presence of MG132 recovered them (Fig. S3), suggesting that proteasomal degradation may be involved in TSA down-regulation of PPARγ isoforms.

### TSA Treatment Attenuates Rosi-mediated Suppression of TNFα-induced ERK Phosphorylation in 3T3-L1 Adipocytes

To elucidate the possible mechanisms underlying TSA attenuation on Rosi-mediated suppression of TNFα-induced lipolysis, downstream signaling pathways of TNFα were examined. Previous studies have identified that p44/42 MAPK (ERK1/2) mediates TNFα-induced lipolysis in adipocytes [7], [12]. Moreover, Rosi treatment suppressed TNFα-induced ERK phosphorylation and lipolysis [12]. Therefore, we tested whether TSA may affect ERK phosphorylation and Rosi-mediated suppression of TNFα-induced ERK phosphorylation.

**Figure 7:**
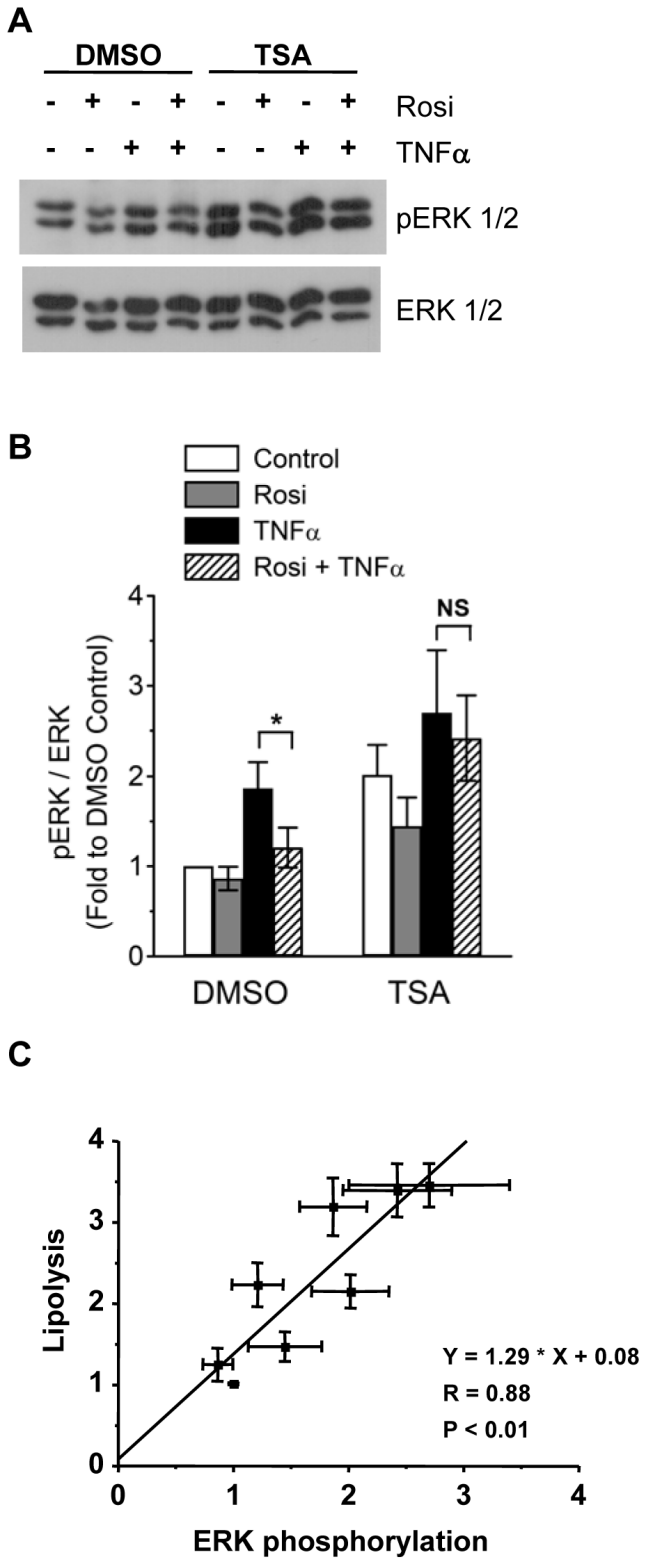
TSA attenuates Rosi-mediated suppression of TNFα-induced ERK1/2 phosphorylation in 3T3-L1 adipocytes. (A and B) 3T3-L1 adipocytes were pretreated with vehicle (DMSO) or 660 nM TSA, together with or without 1 µM Rosi (Rosi) for 24h. Cells were then treated with or without 10 ng/ml TNFα for 30 min. Cellular proteins were solubilized and subjected to SDS-PAGE and Western analysis with the indicated antibodies. Representative immunoblots and quantification data from five independent experiments are shown in 5A and B, respectively. (C) ERK phosphorylation correlates highly with lipolysis in the treatments of Rosi, TNFα, or both in the presence or absence of TSA, as shown by fitting with linear regression. Individual values were obtained from the experiments described in Figures. 1 and 7B.

As shown in Fig. 7A and 7B, TNFα induced ERK phosphorylation, whereas co-treatment with Rosi suppressed TNFα-induced ERK phosphorylation in 3T3-L1 adipocytes, consistent with results from previous reports [12]. Interestingly, TSA treatment alone increased basal ERK phosphorylation, which is consistent with elevated basal lipolysis (Fig. 1). Moreover, the Rosi-mediated suppression of TNFα-induced ERK phosphorylation was attenuated in the presence of TSA (Fig. 7B, TSA, hatched vs black bar). Treatment with MC1568 or MS275 did not affect Rosi-mediated suppression of TNFα-induced ERK phosphorylation, although basal ERK phosphorylation was elevated after MS275 treatment (Fig. S4). These results correlated with the effects of these HDACIs on lipolysis (Fig. 4B). Furthermore, ERK phosphorylation was highly correlated with lipolysis in 3T3-L1 adipocytes measured after the treatments with Rosi, TNFα, or both, in the presence or absence of TSA (Fig. 7C). These results suggest that suppression of ERK phosphorylation may be a mechanism by which Rosi suppresses TNFα action, and TSA may affect Rosi-mediated suppression of TNFα action through modulation of ERK phosphorylation.

### ERK1/2 may Participate in Rosi-mediated Suppression of TNFα-induced Lipolysis and TSA-mediated Attenuation of Rosi Action

**Figure 8:**
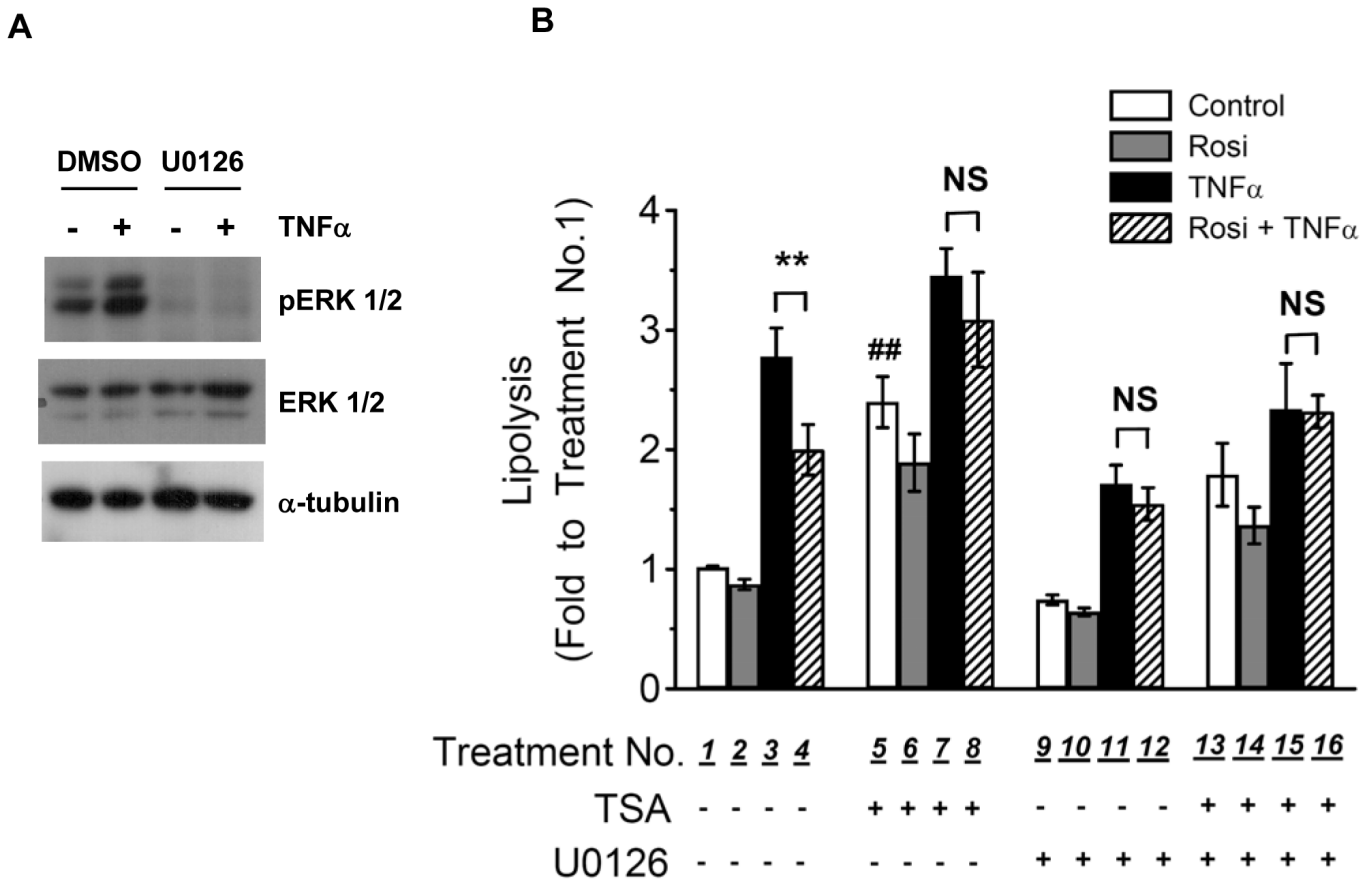
TSA treatment modulates Rosi-mediated suppression of TNFα-induced lipolysis through ERK1/2. (A) 3T3-L1 adipocytes were pretreated with vehicle (DMSO) or 25 µM U0126 for 1 h, followed by treatment with or without 10 ng/ml TNFα for 30 min. Cellular proteins were solubilized and subjected to SDS-PAGE and Western blot analysis with the indicated antibodies. (B) 3T3-L1 adipocytes were treated with vehicle (Control), 1 µM Rosi (Rosi), 10 ng/ml TNFα (TNFα), or both (Rosi+TNFα), together with DMSO (bars 1-4), 660 nM TSA (bars 5-8), 25 µM U0126 (bars 9-12), or both (bars 13-16) for 24h. Glycerol released into the media and protein concentrations of cell lysate were determined as described in *Materials and Methods*. Each point represents the mean ± S.E. of four independent experiments. Asterisks denote significant differences (**: p<0.01). NS, not significant. ^##^p<0.01 vs Treatment No.1.

To determine whether ERK1/2 may participate in TSA attenuation of Rosi-mediated suppression of TNFα-induced lipolysis, we used the inhibitor U0126 of mitogen-activated protein kinase kinase (MEK), the upstream kinase to ERK1/2, in our lipolysis assay. The effectiveness of U0126 as MEK inhibitor was confirmed by Western analysis demonstrating that U0126 treatment abolished TNFα-induced ERK phosphorylation (Fig. 8A). Inhibition of ERK1/2 phosphorylation by U0126 reduced TNFα-induced lipolysis (Fig. 8B, bar 11 vs 3) to a level as Rosi and TNFα co-treatment (Fig. 8B, bar 11 vs 4), suggesting that Rosi suppressed TNFα-induced lipolysis by inhibiting TNFα-induced ERK phosphorylation. Therefore, when ERK phosphorylation was suppressed, Rosi did not reduce TNFα-induced lipolysis further (Fig. 8B, bar 11 vs 12). These results confirmed the role of ERK in the Rosi-mediated suppression of TNFα-induced lipolysis. In the presence of U0126, TSA treatment alone still increased basal lipolysis (Fig. 8B, bar 13 vs 9), and TSA also attenuated Rosi-mediated suppression of TNFα-induced lipolysis (bar 15 vs 16). Because TSA down-regulated PPARγ expression (Fig. 5) and attenuated Rosi-mediated suppression of TNFα-induced ERK phosphorylation (Fig. 7), these results suggested that through modulation of PPARγ level or function, and/or suppression of ERK phosphorylation, TSA attenuated Rosi-mediated suppression of TNFα-induced lipolysis in adipocytes.

## Discussion

Transcription corepressors and HDACs have been shown to mediate the anti-inflammatory actions of TZDs in macrophages [35], [36]. TNFα, a proinflammatory cytokine, chronically stimulates lipolysis in adipocytes, whereas Rosi suppresses TNFα-induced lipolysis. Therefore, we used TSA, a pan HDACI, to test the hypothesis whether HDACs may be involved in Rosi-mediated suppression of TNFα-induced lipolysis in adipocytes. Our results are summarized in Table 1. We have shown that TSA has two effects on adipocyte lipolysis. First, TSA treatment increased basal lipolysis. Second, TSA treatment attenuated the Rosi-mediated suppression of TNFα-induced lipolysis. However, the mechanism by which TSA attenuated the Rosi-mediated suppression of TNFα action may not involve HDAC inhibition. TSA and its related hydroxamate-based HDACI SAHA down-regulated the levels of PPARγ1 and γ2 (Fig. 5), the cellular targets of Rosi, which may account for their attenuation of Rosi-mediated suppression of TNFα-induced lipolysis. Moreover, TNFα-induced lipolysis was in part mediated by phosphorylation of ERK1/2. Rosi suppressed TNFα-induced ERK phosphorylation, thereby inhibiting TNFα-induced lipolysis. MEK inhibitor U0126 inhibited TNFα-induced ERK phosphorylation, and reduced TNFα-induced lipolysis to the same level at which Rosi-mediated suppression, implying that suppression of TNFα-induced ERK phosphorylation may be one of the mechanisms by which Rosi suppresses TNFα-induced lipolysis. TSA, through down-regulation of PPARγ, may attenuate the Rosi-mediated suppression of TNFα-induced ERK phosphorylation and lipolysis. U0126 did not completely abolish TNFα-induced lipolysis (Fig. 8), suggesting that signaling pathways other than ERK1/2 may be involved. Moreover, a PPARγ-independent modulation of ERK phosphorylation and lipolysis by TSA can not be ruled out, which requires further experiments to clarify.

**Table 1:** Summary of the effects of HDACI treatment on lipolysis, ERK phosphorylation, and PPARγ levels.

	TSA	MS275	MC1568
Increased basal lipolysis	+	+	–
Attenuation of Rosi-mediatedsuppression ofTNFα-induced lipolysis	+	–	–
Increased ERK phosphorylation	+	+	–
Attenuation of Rosi-mediatedsuppression ofTNFα-induced ERKphosphorylation	+	–	–
Down-regulation of PPARγ1	+	–	–
Down-regulation of PPARγ2	+	+	–

PPARγ isoforms (γ1 and γ2) are generated by alternative splicing, with PPARγ2 having an additional 30 amino acids in its N terminus. Treatment with broad-spectrum HDACI TSA or SAHA, which inhibits both class I and class II HDACs, down-regulated PPARγ1 and γ2 expression in differentiated adipocytes. The class II HDACI (MC1568) did not affect PPARγ levels, whereas the class I HDACI (MS275) down-regulated PPARγ2 expression to the same level that TSA and SAHA treatment did without affecting PPARγ1 expression (Fig. 5). Previous reports have suggested that PPARγ2 is the more adipogenic PPARγ isoform [39], [40], [41], [42]. However, it is not known whether PPARγ isoforms differ in their anti-inflammatory actions. We found that TSA and MS275 treatment equally down-regulated several known PPARγ target genes such as ATGL, perilipin, G0S2, PDE3B that are involved in adipocyte lipolysis (Fig. S5 and data not shown), consistent with the adipogenic role of PPARγ2 in adipocytes. However, it remains to be elucidated why the sensitivity of PPARγ isoforms to HDACIs is different. Because TSA, but not MS275, attenuated the Rosi-mediated suppression of TNFα-induced ERK phosphorylation and lipolysis, down-regulation of PPARγ2 and these lipolysis-associated PPARγ target genes may not account for the TSA attenuation of the anti-TNFα effects of Rosi. It remains to be determined whether the decrease in total PPARγ levels, the loss of both PPARγ1 and PPARγ2, or other hydroxamate-related but HDACI-independent functions of TSA, may account for the TSA attenuation of TZD-mediated suppression.

Previous reports have shown that during adipocyte differentiation, treatment with 1 mM VPA or 3 nM TSA reduced PPARγ expression, thereby blocking adipogenesis [24]. However in mature adipocytes, treatment with the same dosage of VPA (1 mM) did not affect PPARγ protein levels, and TSA was not included in these studies [37], [38]. In preadipocytes, the TSA concentration that was required for PPARγ down-regulation is much lower (3-10 nM) [24], compared to 660 nM and higher concentrations required for PPARγ down-regulation in differentiated adipocytes (Fig. 6AB), suggesting that preadipocytes are more sensitive to HDACI than differentiated adipocytes. We also observed that even at 2 mM concentration, VPA treatment did not affect Rosi-mediated suppression of TNFα-induced lipolysis or increased basal lipolysis, although it increased hyperacetylation of histone H3 in adipocytes (Fig. S6). It is possible that the VPA concentration required for down-regulation of PPARγ2 in adipocytes may be higher. Alternatively, structurally different HDACIs may have different effects on PPARγ expression levels in adipocytes.

Phosphorylation of ERK1/2 has been shown to mediate lipolysis induced by many chronic stimuli, such as ER stress [43], Fas ligand [44], interleukin-6 [45], lipopolysaccharide [46], and TNFα [7], [12], as indicated by the fact that MEK inhibitor treatment (U0126 or PD98059) attenuates lipolysis induced by these chronic stimuli. Inhibition of ERK phosphorylation has also been correlated with pharmacological suppression of chronic lipolysis. For example, salicylate [47] and metformin [48] treatment suppress TNFα-induced ERK phosphorylation and lipolysis, whereas Rosi treatment suppresses ERK phosphorylation and lipolysis induced by Fas ligand [44], interleukin-6 [45], and TNFα [8], [12]. These results suggest that ERK1/2 is an important regulator of chronically stimulated lipolysis. However, the mechanisms by which different pharmacological inhibitors suppress ERK phosphorylation remains to be elucidated. Our results also imply that ERK1/2 is a molecular target for Rosi-mediated suppression and TSA-mediated modulation of TNFα-induced lipolysis (Fig. 7 and 8). Many upstream kinases or phosphatases may be potential targets for Rosi or TSA modulation to affect ERK phosphorylation in adipocytes. For example, MAP3K8 (also called Tpl2), an upstream kinase to ERK, is up-regulated in obese adipocytes and mediates TNFα-induced ERK activation and lipolysis [49]. MAP kinase phosphatases 1 and 4 have been reported to play a role in pathogenesis of insulin resistance and adipocyte hypertrophy [50], [51]. Protein phosphatases such as PP5, have been reported to modulate PPARγ phosphorylation and function [52]. Further experiments will be required to determine whether these upstream kinases and phosphatases are involved in Rosi-mediated suppression and TSA-mediated modulation of TNFα-induced ERK activation and lipolysis.

Many histone acetylation-independent effects have been reported for HDACIs [17]. In glioblastoma and prostate cancer cells, TSA and SAHA but not MS275 targeted HDAC1 and 6, thereby disrupting the HDAC and protein phosphatase 1 complex. The release of the protein phosphatase from the HDACs resulted in decreased phosphorylation of Akt and ERK1/2 [53]. However, we did not observe changes in Akt phosphorylation after TSA treatment (data not shown), thus ruling out that decreased Akt activity mediated increases in lipolysis [54]. Moreover, phosphorylation of ERK1/2 was increased after TSA treatment in our experiments (Fig. 6). These results suggest that other mechanisms may account for the TSA-mediated effects in our system.

HDAC inhibitors have emerged as a potential therapeutic method for a wide range of diseases, including cancer, inflammatory, and cardiovascular diseases [17], [55]. Although clinically they are well tolerated in general, they do have side effects, and the molecular mechanisms underlying these side effects remains elusive. Metabolic effects of HDACI treatment have been reported, such as weight gain and insulin resistance for VPA [21], [23]. Moreover, HDACI treatment can also alter cellular metabolism, such as fatty acid oxidation and glucose metabolism [22]. The present studies have shown that hydroxamate-based HDACIs, including TSA and SAHA, may affect adipocyte functions such as lipolysis and modulate the anti-TNFα action of TZDs through PPARγ. In light of the clinical application of SAHA and many other HDACIs, a better understanding of their cellular mechanisms and interactions with other therapeutic drugs may allow the design of more effective strategies in disease treatment.

## Supporting Information

Figure S1 **Depletion of endogenous SMRT, NCoR, or PPARγ in 3T3-L1 adipocytes by RNAi.** 3T3-L1 adipocytes were transfected with non-targeting luciferase siRNA (Luc) or siRNA against SMRT, NCoR, or PPARγ. Cellular proteins were solubilized and subjected to SDS-PAGE and Western blot analysis with the indicated antibodies.
(TIF)

Figure S2 **Dose- and time-dependent down-regulation of PPARγ1 and γ2 by TSA treatment.** (A, B) 3T3-L1 adipocytes were treated in duplicate with increasing TSA doses (0, 6.6, 66, 660, 6600 nM) for 24 h. Cellular proteins were solubilized and subjected to SDS-PAGE and Western blot analysis. Quantification data for PPARγ1 and γ2 from three independent experiments are shown in Fig. S2A and B, respectively. Asterisks denote significant differences compared with 0 nM control (*p<0.05; ***p<0.001). (C, D) 3T3-L1 adipocytes were treated with vehicle (DMSO) or 660 nM TSA (TSA) for 0, 1, 2, 6, 12, or 24 h. Cellular proteins were solubilized and subjected to SDS-PAGE and Western blot analysis. Quantification data for PPARγ1 and γ2 from four independent experiments are shown in Fig. S2C and D, respectively. Asterisks denote significant differences compared with corresponding DMSO value at the same time point (*p<0.05; ***p<0.001).
(TIF)

Figure S3 **Treatment of proteasomal inhibitor reverses TSA down-regulation of PPARγ.** (A) 3T3-L1 adipocytes were treated with vehicle (Control), 660 nM TSA, 20 µM MG132, or both for 6 hr. Cellular proteins were solubilized and subjected to SDS-PAGE and Western blot analysis with the indicated antibodies. Representative immunoblots from four independent experiments were shown in Fig. S3A. (B-D) Quantification data for total PPARγ (B), PPARγ1 (C), and PPARγ2 (D) are shown. Asterisks denote significant differences (p<0.05).
(TIF)

Figure S4 **MS275 or MC1568 treatment does not affect Rosi-mediated suppression of TNFα-induced ERK phosphorylation.** 3T3-L1 adipocytes were pretreated with vehicle (DMSO), 5 µM MC1568, or 10 µM MS275, together with or without 1 µM Rosi (Rosi) for 24h. Cells were then treated with or without 10 ng/ml TNFα for 30 min. Cellular proteins were solubilized and subjected to SDS-PAGE and Western blot analysis with the indicated antibodies. Representative immunoblots are shown.
(TIF)

Figure S5 **Gene expression levels after TSA treatment.** 3T3-L1 adipocytes were treated with vehicle (Control), 1 µM Rosi (Rosi), 10 ng/ml TNFα (TNFα), or both (Rosi+TNFα), together with vehicle (DMSO) or 660 nM TSA (TSA) for 24 h. The levels of mRNA were determined by qPCR. Each point represents the mean ± S.E. of at least three independent experiments. Asterisks denote significant differences (*p<0.05; ***p<0.001). ^#^p<0.05; ^##^p<0.01; ^###^p<0.001 compared with corresponding DMSO control.
(TIF)

Figure S6 **VPA does not affect the Rosi-mediated suppression of TNFα-induced lipolysis.** (A) 3T3-L1 adipocytes were treated in duplicate with vehicle (H_2_O) or 2 mM VPA for 24 h. Cellular proteins were solubilized and subjected to SDS-PAGE and Western blot analysis with the indicated antibodies. Representative immunoblots are shown. (B) 3T3-L1 adipocytes were treated with vehicle (Control), 1 µM Rosi (Rosi), 10 ng/ml TNFα (TNFα), or both (Rosi+TNFα), together with vehicle or 2 mM VPA for 24 h. Glycerol released into the media and protein concentrations of cell lysate were determined as described in *Materials and Methods*. Each point represents the mean ± S.E. of three independent experiments. Asterisks denote significant differences (***p<0.001).
(TIF)
