# Supplementary figures and images for: Trichostatin A Modulates Thiazolidinedione-Mediated Suppression of Tumor Necrosis Factor α-Induced Lipolysis in 3T3-L1 Adipocytes
Source: PLoS One. 2013 Aug 9;8(8):e71517. doi: 10.1371/journal.pone.0071517 (PMC3739734; doi:10.1371/journal.pone.0071517)

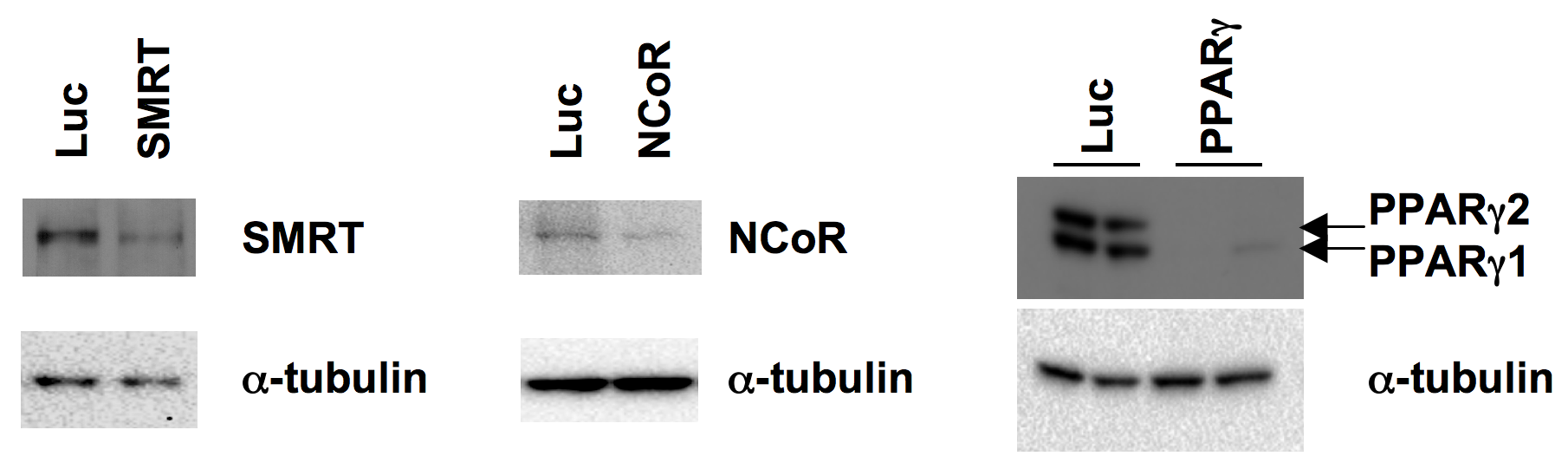

Supplement: Figure S1 — Depletion of endogenous SMRT, NCoR, or PPARγ in 3T3-L1 adipocytes by RNAi. 3T3-L1 adipocytes were transfected with non-targeting luciferase siRNA (Luc) or siRNA against SMRT, NCoR, or PPARγ. Cellular proteins were solubilized and subjected to SDS-PAGE and Western blot analysis with the indicated antibodies. (TIF) [file pone.0071517.s001.tif]

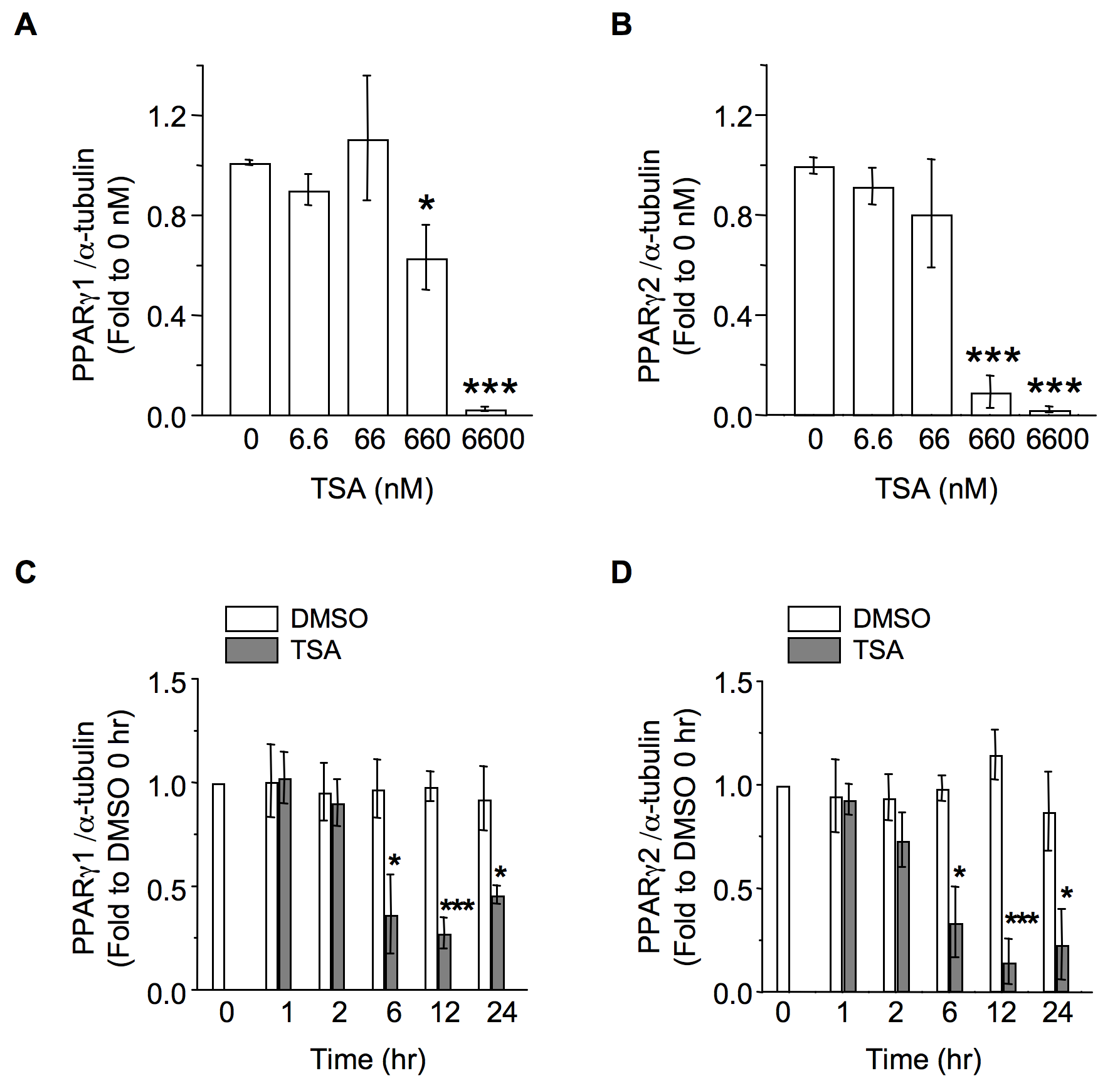

Supplement: Figure S2 — Dose- and time-dependent down-regulation of PPARγ1 and γ2 by TSA treatment. (A, B) 3T3-L1 adipocytes were treated in duplicate with increasing TSA doses (0, 6.6, 66, 660, 6600 nM) for 24 h. Cellular proteins were solubilized and subjected to SDS-PAGE and Western blot analysis. Quantification data for PPARγ1 and γ2 from three independent experiments are shown in Fig. S2A and B, respectively. Asterisks denote significant differences compared with 0 nM control (*p<0.05; ***p<0.001). (C, D) 3T3-L1 adipocytes were treated with vehicle (DMSO) or 660 nM TSA (TSA) for 0, 1, 2, 6, 12, or 24 h. Cellular proteins were solubilized and subjected to SDS-PAGE and Western blot analysis. Quantification data for PPARγ1 and γ2 from four independent experiments are shown in Fig. S2C and D, respectively. Asterisks denote significant differences compared with corresponding DMSO value at the same time point (*p<0.05; ***p<0.001). (TIF) [file pone.0071517.s002.tif]

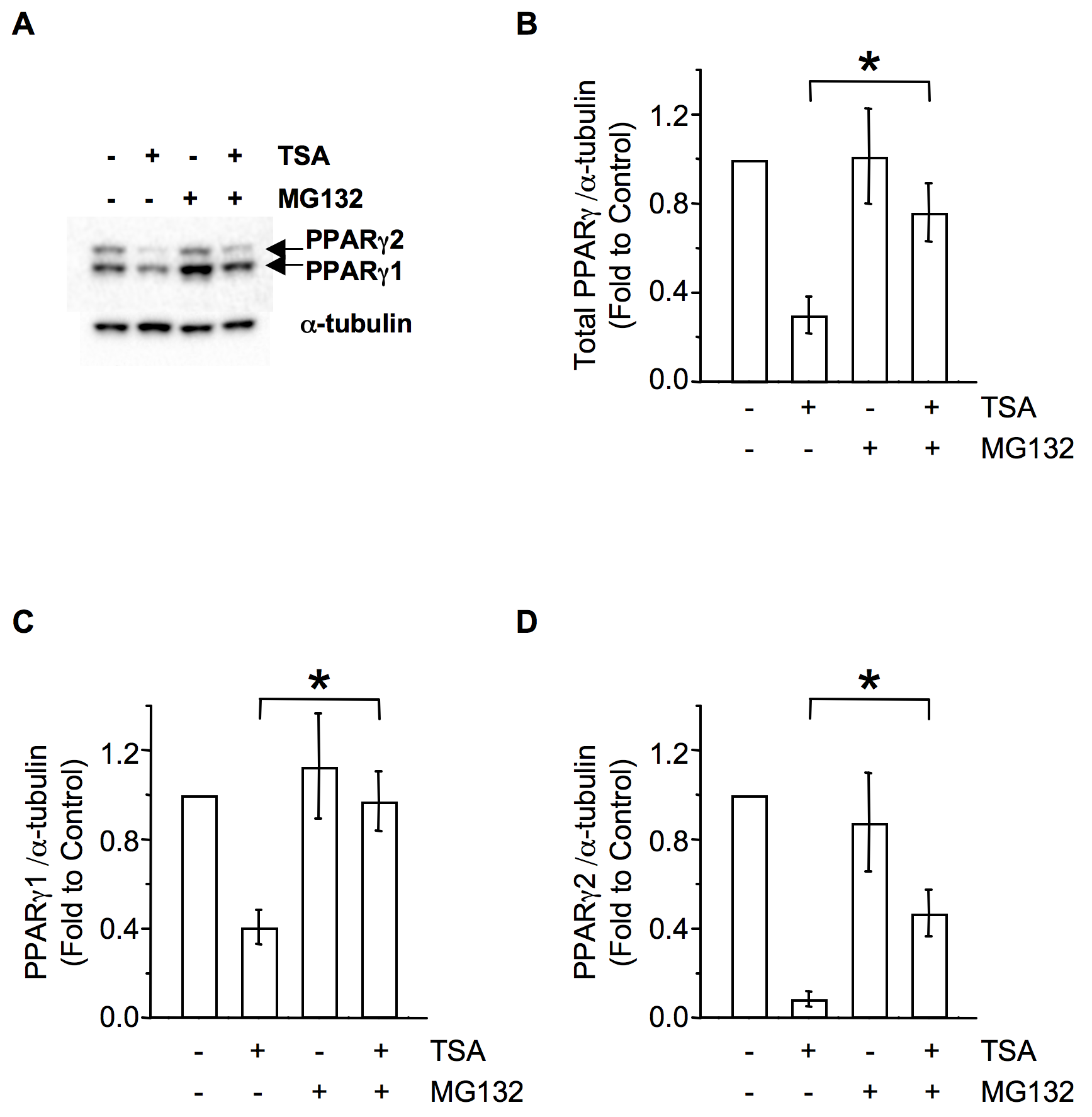

Supplement: Figure S3 — Treatment of proteasomal inhibitor reverses TSA down-regulation of PPARγ. (A) 3T3-L1 adipocytes were treated with vehicle (Control), 660 nM TSA, 20 µM MG132, or both for 6 hr. Cellular proteins were solubilized and subjected to SDS-PAGE and Western blot analysis with the indicated antibodies. Representative immunoblots from four independent experiments were shown in Fig. S3A. (B-D) Quantification data for total PPARγ (B), PPARγ1 (C), and PPARγ2 (D) are shown. Asterisks denote significant differences (p<0.05). (TIF) [file pone.0071517.s003.tif]

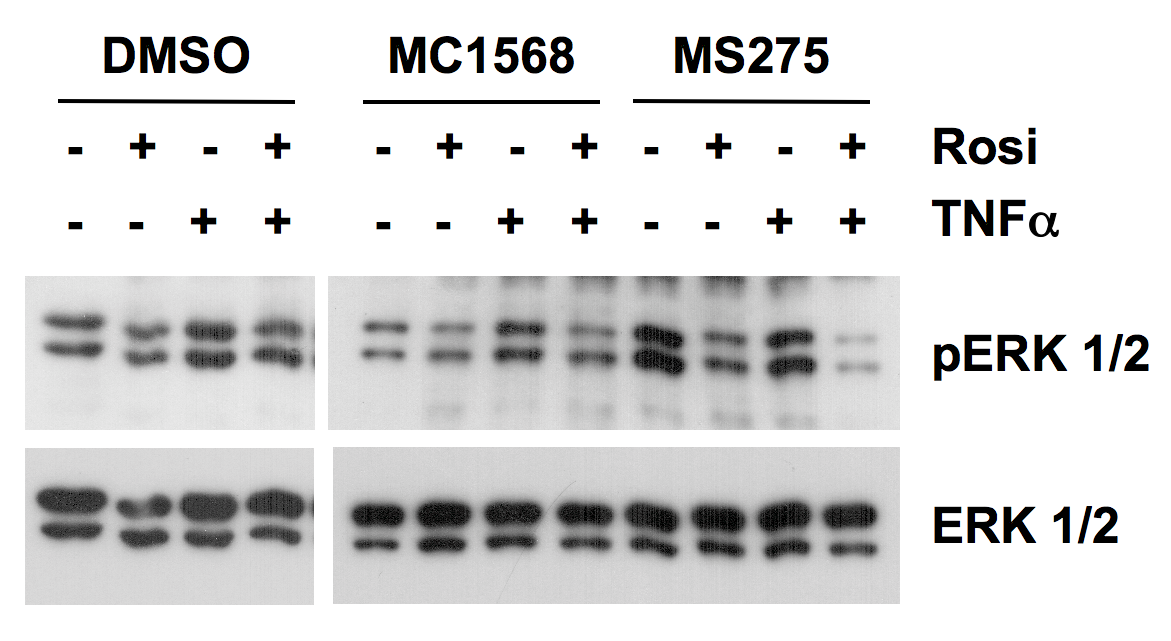

Supplement: Figure S4 — MS275 or MC1568 treatment does not affect Rosi-mediated suppression of TNFα-induced ERK phosphorylation. 3T3-L1 adipocytes were pretreated with vehicle (DMSO), 5 µM MC1568, or 10 µM MS275, together with or without 1 µM Rosi (Rosi) for 24h. Cells were then treated with or without 10 ng/ml TNFα for 30 min. Cellular proteins were solubilized and subjected to SDS-PAGE and Western blot analysis with the indicated antibodies. Representative immunoblots are shown. (TIF) [file pone.0071517.s004.tif]

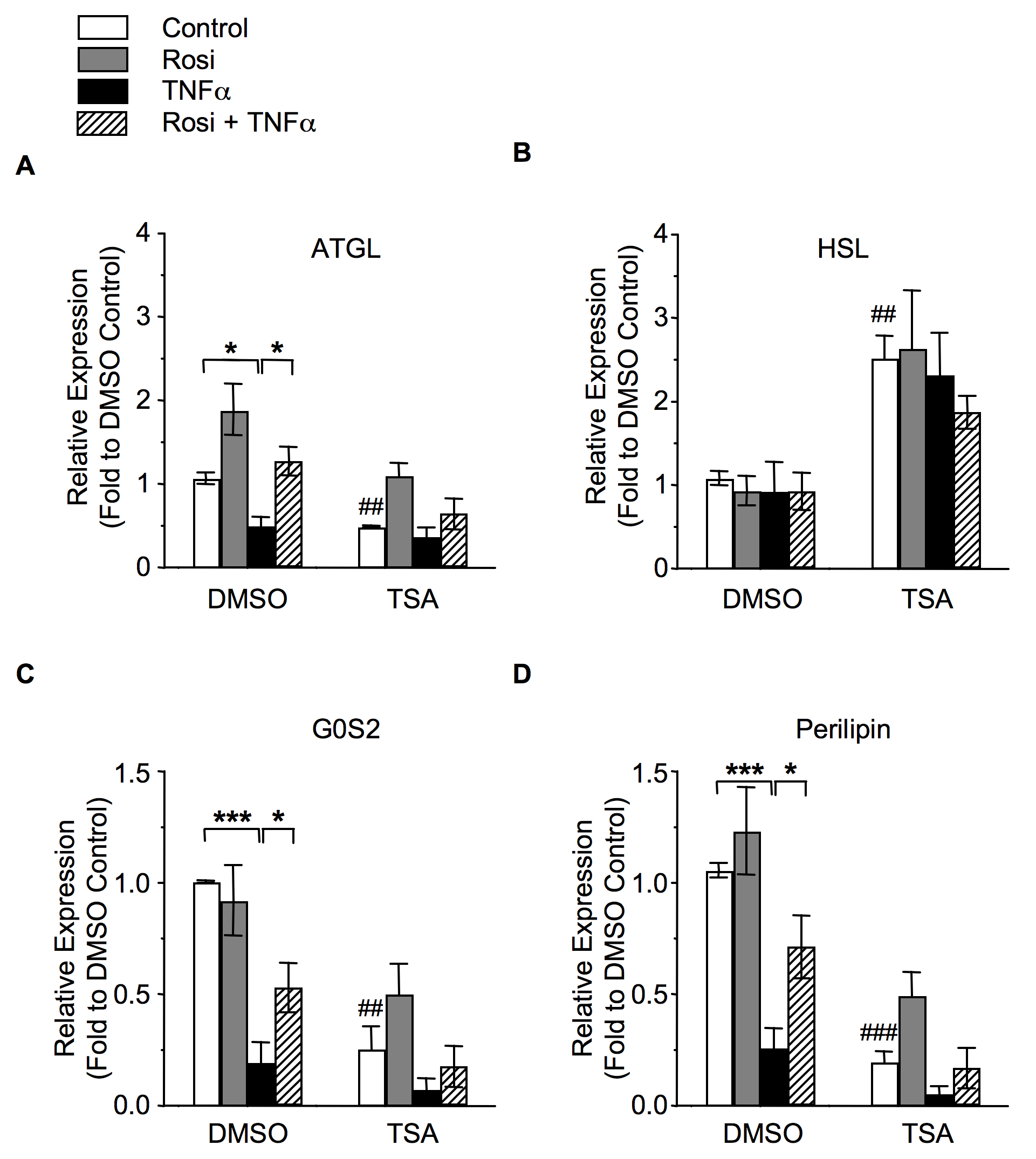

Supplement: Figure S5 — Gene expression levels after TSA treatment. 3T3-L1 adipocytes were treated with vehicle (Control), 1 µM Rosi (Rosi), 10 ng/ml TNFα (TNFα), or both (Rosi+TNFα), together with vehicle (DMSO) or 660 nM TSA (TSA) for 24 h. The levels of mRNA were determined by qPCR. Each point represents the mean ± S.E. of at least three independent experiments. Asterisks denote significant differences (*p<0.05; ***p<0.001). #p<0.05; ##p<0.01; ###p<0.001 compared with corresponding DMSO control. (TIF) [file pone.0071517.s005.tif]

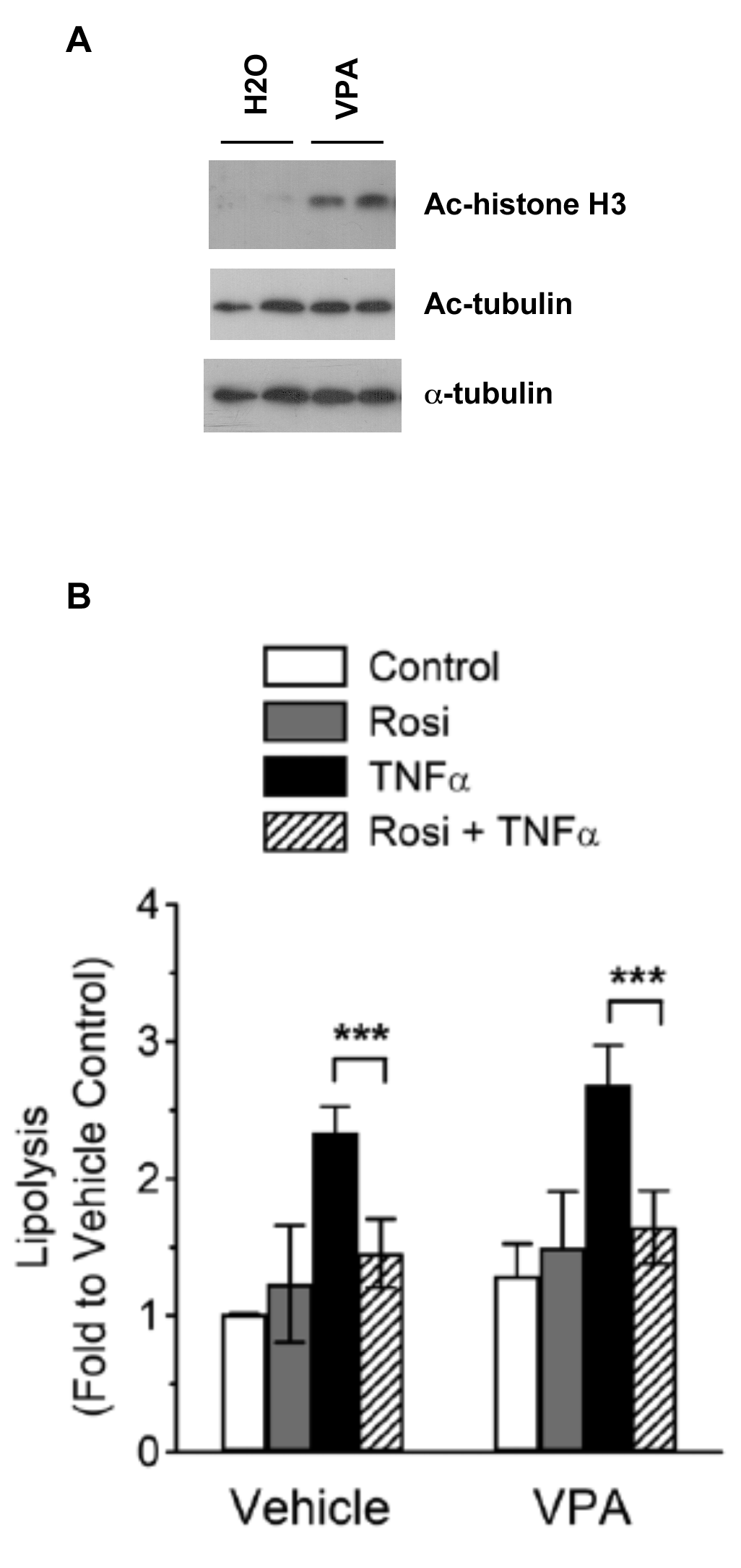

Supplement: Figure S6 — VPA does not affect the Rosi-mediated suppression of TNFα-induced lipolysis. (A) 3T3-L1 adipocytes were treated in duplicate with vehicle (H2O) or 2 mM VPA for 24 h. Cellular proteins were solubilized and subjected to SDS-PAGE and Western blot analysis with the indicated antibodies. Representative immunoblots are shown. (B) 3T3-L1 adipocytes were treated with vehicle (Control), 1 µM Rosi (Rosi), 10 ng/ml TNFα (TNFα), or both (Rosi+TNFα), together with vehicle or 2 mM VPA for 24 h. Glycerol released into the media and protein concentrations of cell lysate were determined as described in Materials and Methods. Each point represents the mean ± S.E. of three independent experiments. Asterisks denote significant differences (***p<0.001). (TIF) [file pone.0071517.s006.tif]
